# Exercise intensity and reliability during recreational team handball training for 50–77-year-old unexperienced women

**DOI:** 10.5114/biolsport.2024.132995

**Published:** 2024-05-17

**Authors:** Rita Pereira, Peter Krustrup, Carlo Castagna, Carlos Resende, Ivone Carneiro, José Magalhães, Susana Póvoas

**Affiliations:** 1 Laboratory of Metabolism and Exercise (LaMetEx), Research Centre in Physical Activity, Health and Leisure (CIAFEL), Faculty of Sport, University of Porto, Porto, Portugal; 2 University of Maia, Maia, Portugal; 3 Department of Sports Science and Clinical Biomechanics, SDU Sport and Health Sciences Cluster (SHSC), University of Southern Denmark, Odense, Denmark; 4 Danish Institute for Advanced Study (DIAS), University of Southern Denmark, Odense, Denmark; 5 Sport and Health Sciences, University of Exeter, Exeter, UK; 6 Shanghai University of Sport (SUS), Shanghai, China; 7 Department of Biomolecular Sciences, School of Exercise and Health Sciences, Carlo Bo Urbino University, Urbino, Italy; 8 Centre of Research, Education, Innovation and Intervention in Sport (CIFID), Faculty of Sport, University of Porto, Porto, Portugal; 9 Research Center in Sports Sciences, Health Sciences and Human Development (CIDESD), University of Maia, Maia, Portugal

**Keywords:** Team sports, Intermittent training, Activity profile, Global positioning system, Cardiovascular response

## Abstract

This study analyzed the physiological and physical demands of recreational team handball (RTH) and the training sessions’ (matches) intensity variability in 50–77-year-old postmenopausal women (n = 20) without prior experience with the sport. Heart rate (HR), blood lactate, rating of perceived exertion (RPE), distance covered, time spent in different locomotor categories, accelerations, decelerations, Player Load (PL), game actions and fun levels were evaluated in 245 matches, played indoor (n = 130) or outdoor (n = 115), as small-sided games (3 × 15-min periods). Mean and peak HRs were 79 and 88% of maximal HR (%HR_max_), showing reliable values across the training sessions, with time spent in the HR zones showing excellent relative reliability, though poor absolute reliability. Time spent > 80 and > 90%HR_max_ was 48% and 14% of total match time, respectively. Mean and peak blood lactate values were 2.6 ± 0.8 and 2.9 ± 0.9 mmol · l^−1^, respectively. RPE was 5.5 ± 1.5, showing good relative though poor absolute reliability, and fun levels were 8.4 ± 1.1 (0–10 scale). Total distance covered was 1878 ± 333 m and the participants spent 54%, 3% and < 1% of total match time walking, fast running and sprinting, respectively. Total PL was 224 ± 41 (AU), with 67% of total match time being spent in low-intensity zones. Participants performed a total of 38 specific high-demanding actions per match, mainly throws (10.6 ± 5.8) and stops (11.0 ± 3.6). RTH training, played as small-sided games, is a high-intensity exercise training mode with high aerobic and anaerobic demands, low RPE and high fun levels for 50–77-year-old women without prior experience with the sport. Average cardiovascular demands were consistent across the training sessions.

## INTRODUCTION

Although the unequivocal positive health effects of physical activity (PA) and exercise, physical inactivity levels in Europe are high, particularly in over 55-year-old men and women [1]. Lack of motivation and interest is one of the main barriers to exercise or play sport, while enjoyment and fun have been reported as motives for being physically active [1, 2]. Thus, the World Health Organization (WHO) has made a call for innovative, health-enhancing exercise options, highlighting the role of sport in counteracting physical inactivity, and improving health by setting promotion of sport for all as a central policy recommendation in the WHO Global Action Plan on PA 2018–2030 [3]. In fact, team sports seem to meet the population’s interests, based on the estimated high number of participants [4]. Football, team handball (TH), floorball, basketball, touch rugby and futsal have been adapted to its recreational versions (i.e., involving lower number of players, smaller areas, limited body contact, simpler rules) to be used in exercise interventions aiming at health enhancements [4]. Team sports are an example of multicomponent exercise modalities, casuistically and intermittently combining low to high-intensity locomotion activities and high-demanding specific actions (e.g., jumps, accelerations, decelerations, changes of direction and stops) [4]. Therefore, the physical demands of team sports are aligned with the PA guidelines for adults and older adults that focus on moderate to vigorous-intensity aerobic PA, muscle-strengthening activities and balance training, to improve cardiorespiratory and muscular fitness, bone health, functional balance and preventing falls [5]. Exercise interventions using recreational team sports usually entail 2 weekly sessions of 40–60 min (i.e., 80–120 min/week), with mean heart rates (HRs) ranging from 71–85% of maximal HR (HR_max_) [4]. Hence, team sports practice under these conditions (volume and intensity) meets part of the PA guidelines [5]. Moreover, ball games and team sports have been reported to be a motivating exercise mode [6], thus counteracting one of the main barriers to exercise [1, 2].

Recreational team handball (RTH) has shown to induce several health benefits, namely by improving cardiovascular, bone and metabolic health and physical fitness of different populations [7–12]. Particularly in postmenopausal women, RTH practice showed to improve peak oxygen uptake (VO_2peak_), aerobic performance, bone mineral density (BMD) and content (BMC), bone turnover markers, postural balance, body mass, body fat, and total and low-density lipoprotein cholesterol [11, 12]. This is a population of interest considering the deleterious broad-spectrum impact of menopause on women’s health [13], and that physical inactivity levels increase with age, and are higher in women than in men [1]. Nonetheless, even though the health benefits of RTH practice in this population have been described, its working demands have not been addressed in detail so far. Information on the external and internal load experienced during small-sided RTH games will result of great practical interest as it may explain the reasons underlying the observed health adaptations and allow for training prescription accuracy. Moreover, due to the casuistic nature of this exercise modality, it is of importance to know how the cardiovascular stress (HR) varies between training sessions that are based on RTH matches.

Therefore, the aim of this study is to examine the physiological (internal load) and physical demands (external load) induced by the most frequently used small-sided RTH games (i.e., 4 v 4 to 6 v 6) in postmenopausal women without prior experience with the sport and to analyze the intensity (HR) variability between training sessions (matches). Based on the results of previous studies [14, 15], we hypothesized that small-sided RTH games are a vigorous exercise activity, comprising high-intensity specific actions able to improve the cardiovascular and musculoskeletal systems of postmenopausal women. We also hypothesized that there is variability in the intensity (HR) among training sessions, although the cardiovascular stress is high on average, providing stimulus in the range to induce positive cardiovascular health adaptations.

## MATERIALS AND METHODS

### Participants

Recruitment started one month before the beginning of the trial. Twenty 50–77-year-old postmenopausal women (mean age 64 ± 7 years, stature 158 ± 7 cm, body mass 65.5 ± 10.9 kg, fat mass 36.0 ± 6.4%, VO_2peak_ 25.7 ± 3.4 mL · min · kg^−1^) volunteered to participate in this study. Inclusion criteria were: women with menopause for at least 3 years and inactive (i.e., not complying with the PA guidelines for the last 6 months). Exclusion criteria were: participants that presented any contraindication to perform moderate-to-vigorous PA or incapacity to run or grip a ball.

All participants were informed verbally and in written about the purpose, potential risks, and benefits of the study and that they could withdraw at any time without penalty and signed a written informed consent. The study was carried out in accordance with the Declaration of Helsinki and ethical approval was provided by the local Ethics Committee (CEFADE 20 2019).

### Experimental design

Internal and external load markers and fun levels evaluations were performed at the beginning of a RTH-based exercise intervention. Detailed description of the study is provided elsewhere [11, 12]. The participants randomly performed RTH matches (played as 4 v 4, 5 v 5 and 6 v 6), preceded by a standardized warm-up, in indoor (n = 130) and outdoor conditions (n = 115), organized in 3 × 15-min periods interspersed by 2-min breaks, and with at least 48 h of rest between matches. The matches were carried out on a regular TH court (40 × 20 m) adjusted in length and width to result in 34–36 m^2^/player. The participants were hydrated at the beginning of the session and were allowed to drink water *ad libitum* to ensure the maintenance of proper hydration.

The participants rotated positions every 3 min in a random order, including the goalkeeper, and no exclusions or substitutions were applied. Dribbling was not allowed, and the ball was immediately put back in play by the goalkeeper after a goal. To avoid injuries, no hard tackles and physical contact were permitted, and the balls were made of soft material and lighter than the official ones.

The physiological and physical demands were analyzed considering internal [HR, blood lactate (BL), rating of perceived exertion (RPE)] and external [distance covered and time spent in different locomotor categories, accelerations, decelerations, Player Load (PL), and specific game actions] load variables and fun levels were also determined. In indoor conditions, only HR, RPE and fun levels were assessed, while in outdoor conditions all the study variables were evaluated. No differences were observed between indoor and outdoor conditions in mean and peak HR, time spent in the HR zones, RPE and fun levels (data not shown) and therefore, the data from the matches performed in these two conditions were analyzed together.

### Experimental procedures

The participants were familiarized with all the procedures before the data collection. HR was evaluated using HR monitors (Firstbeat Technologies Ltd., version 4.7.2.1., Jyväskylä, Finland). Mean and peak HR are expressed as absolute (b · min^−1^) and relative values (%HR_max_) and individual HR_max_ was determined according to a multiple testing approach [16]. HR zones were ≤ 60, 61–70, 71–80, 81–90 and 91–100% of individual HR_max_. Capillary blood samples (~30 μL) were collected from the right earlobe at rest (baseline) and randomly in each match period, using a portable electroenzymatic lactate device analyzer (Lactate Pro 2 LT-1730, Arkray, Amsterdam, The Netherlands) to access BL concentrations (mmol · l^−1^). RPE (0–10, AU, Borg scale) [17] and fun levels (0–10, AU, visual analogic scale) [18] were evaluated at the end of the training sessions.

Distance covered (m) and time spent (s) in different locomotor categories (standing, walking, jogging, fast running and sprinting), accelerations and decelerations, and PL were measured using global positioning system (GPS) units (Catapult MinimaxX S4; Catapult Sports, Canberra, Australia), following standardized procedures. Catapult Sprint software (Catapult Innovations, Canberra, Australia, version 5.1.1) was used to export the data. The validity and reliability of the accelerometers have been described elsewhere [19]. In this study, individual speed thresholds were considered to normalize external load variables. For every participant, the mean velocity of each locomotor category over a set distance (20 m) was determined using telemetric photoelectric cells (Brower Timing System, IRD-T175, UT, USA). The mean velocity of the participants in each category was 6, 8, 12 and 13 km · h^−1^ for walking, jogging, fast running and sprinting, respectively. The resulting scores were used to set individual thresholds for the considered arbitrary speed categories. PL (estimated by triaxial accelerometry, measured at a 100 Hz sampling rate) was presented as total PL (AU) and percentage of time spent in PL zones (< 0.1, 0.1–0.3, 0.3–0.6, 0.6–1.0, 1.0–1.5, 1.5–2.0, > 2.0). The number of accelerations and decelerations was determined and categorized as low (1.50–2.14 m · s^−1^), medium (2.14–2.78 m · s^−1^) and high-intensity (> 2.78 m · s^−1^), according to manufacture settings (Catapult Sprint Version 5.1.1 software manual, Catapult Innovations, Canberra, Australia). Individual workload (Kg · m) was calculated multiplying the total distance covered by the body mass. Qualitative analyses were performed using video recordings (SONY-DCR-SX65E, digital video camera recorder, UK) to account for game actions, namely jumps, throws, stops, changes of direction, and one-on-one situations. The external load data [i.e., total distance covered (ICC 0.59; 0.30–0.80), total PL (ICC 0.69; 0.44–0.86), time spent in fast running (ICC 0.84; 0.67–0.93) and jogging (ICC 0.75; 0.53–0.89)] demonstrated good to excellent relative reliability. The matches were held under neutral temperature (17–22°C) and humidity (50–70%) conditions.

### Statistics

Data are presented as mean ± standard deviations (SD). Differences in internal and external load variables between the three periods were assessed by one-way repeated measures analysis of variance (ANOVA) with Bonferroni post hoc test. Differences in HR variables, RPE and fun in indoor and outdoor conditions were assessed by Student’s paired *t*-test. Practical significance was assessed by calculating Cohen *d* and interpreted as trivial (< 0.2), small (0.2–0.5), medium (0.5–0.8) and large (> 0.8) [20]. Mean and peak HR and HR zones’ absolute reliability was assessed using the typical error of measurement (TEM) and expressed as raw and as percentage of the coefficient of variation (%CV) in 3 randomly selected matches. Random errors were interpreted as good, moderate and modest to poor, if %CV and TEM as %CV were ≤ 10%, > 10-20% and >20%, respectively [21]. The intraclass correlation coefficient (ICC_3,1_) was used to calculate relative reliability [22, 23], with values 0.75–1.00 being considered as excellent, 0.41–0.74 as good, and 0.00–0.40 as poor [24]. Statistical Package for the Social Sciences (SPSS Inc., version 25) was used for data analysis. Statistical significance was set at *p* ≤ 0.05.

## RESULTS

### Internal load variables and fun levels

Mean and peak HR and time spent in the HR zones during the RTH matches held in indoor and outdoor conditions are presented in Table 1. Mean HR increased from the first to the second period (*p* = 0.002; *d* = 0.916). Peak HR in the second period was higher than in the first (*p* = 0.025; *d* = 0.662) and third periods (*p* = 0.005; *d* = 0.799). Time spent ≤ 60%HR_max_ decreased from the first to the second (*p* = 0.002; *d* = 1.062) and third periods (*p* = 0.034; *d* = 0.682). No differences were found between the match periods in time spent in any other HR zones.

**TABLE 1:** Heart rate and time spent in selected heart rate zones during each match period and total match duration of recreational team handball matches (mean ± SD)

Variables	1^st^ period	2^nd^ period	3^rd^ period	Total match
Mean heart rate (b · min^−1^)	134 ± 15	137 ± 15^*^	136 ± 15	136 ± 15
Mean heart rate (%HR_max_)	77.8 ± 5.4	79.6 ± 5.5^*^	79.0 ± 5.8	78.8 ± 5.4
Peak heart rate (b · min^−1^)	151 ± 14	153 ± 15^*^	151 ± 15^#^	152 ± 14
Peak heart rate (%HR_max_)	87.5 ± 4.2	88.7 ± 4.7^*^	87.4 ± 5.0^#^	87.9 ± 4.5
Time ≤ 60%HR_max_ (%)	5.6 ± 6.3	2.8 ± 4.7^*^	2.9 ± 4.5^*^	3.8 ± 5.0
Time 61–70%HR_max_ (%)	18.7 ± 15.1	15.8 ± 12.6	17.1 ± 15.7	17.1 ± 13.5
Time 71–80%HR_max_ (%)	30.0 ± 13.1	32.2 ± 13.7	32.4 ± 13.9	31.5 ± 12.6
Time 81–90%HR_max_ (%)	33.0 ± 17.3	34.0 ± 16.6	34.0 ± 18.0	33.7 ± 16.2
Time 91–100%HR_max_ (%)	13.1 ± 16.7	15.8 ± 18.5	14.2 ± 16.5	14.4 ± 16.9
Time > 80%HR_max_ (%)	46.1 ± 26.6	49.8 ± 25.3	48.2 ± 28.7	48.1 ± 25.7

* Significantly different from the first period.

# Significantly different from the second period.

**FIG. 1:**
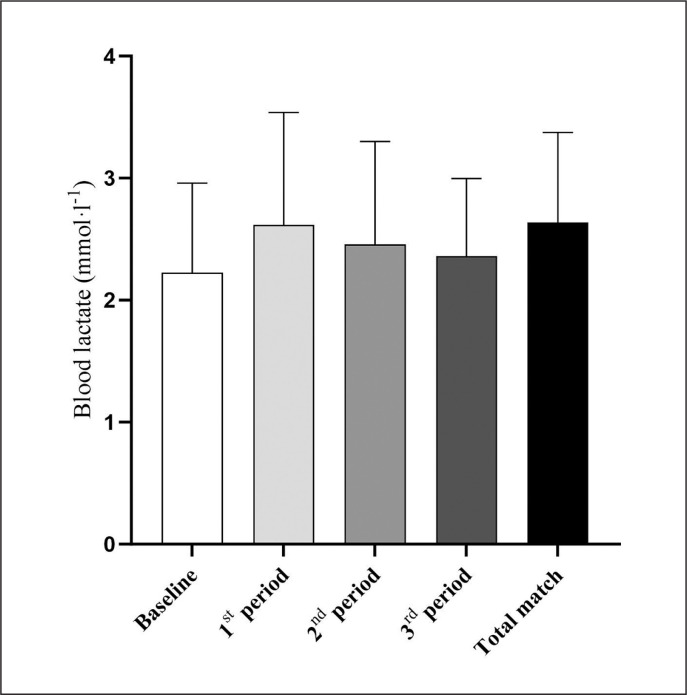
Mean blood lactate at baseline and during the first, second and third periods and total match duration during recreational team handball matches.

Baseline values and mean and peak BL values for the total match time during the RTH matches performed outdoors were 2.2 ± 0.7, 2.6 ± 0.8 and 2.9 ± 0.9 mmol · l^−1^, respectively, with peak values being higher compared to baseline (*p* = 0.047; *d* = 0.844) and mean values (*p* = 0.001; *d* = 1.717). Mean BL was 2.6 ± 0.9, 2.4 ± 0.8 and 2.4 ± 0.6 mmol · l^−1^ for the first, second and third periods, respectively, with no differences between them and with baseline (Figure 1). Fun levels were reported as 8.4 ± 1.1 and RPE was 5.5 ± 1.5 (0–10, AU), during the RTH matches held in indoor and outdoor conditions.

### External load variables

Distance covered, accelerations and decelerations, PL and game actions data were evaluated in outdoor matches.

Time spent and distance covered in each locomotor category, total distance covered, and individual workload are presented in Table 2. There were no differences between the match periods regarding these variables.

**TABLE 2:** Time spent and distance covered in different locomotor categories, total distance covered and individual workload during each match period and total match duration of recreational team handball matches (mean ± SD)

Variables	1^st^ period	2^nd^ period	3^rd^ period	Total match
Standing				
Time (s)	348 ± 81	351 ± 79	379 ± 97	1078 ± 226
Time (%)	38 ± 9	38 ± 9	40 ± 10	38 ± 8

Walking				
Distance (m)	458 ± 79	464 ± 63	458 ± 77	1380 ± 197
Distance (%)	76 ± 9	76 ± 9	77 ± 9	76 ± 9
Time (s)	504 ± 74	509 ± 65	502 ± 79	1516 ± 196
Time (%)	55 ± 8	55 ± 7	53 ± 8	54 ± 7
Individual workload (Kg·m)	29468 ± 5511	29939 ± 5283	29663 ± 6570	89071 ± 16163

Jogging				
Distance (m)	93 ± 48	92 ± 53	89 ± 53	274 ± 148
Distance (%)	14 ± 7	14 ± 7	13 ± 7	14 ± 7
Time (s)	46 ± 24	46 ± 25	44 ± 26	135 ± 72
Time (%)	5 ± 3	5 ± 3	5 ± 3	5 ± 3
Individual workload (Kg·m)	5981 ± 3243	5931 ± 3501	5797 ± 3493	17709 ± 9900

Fast running				
Distance (m)	66 ± 65	67 ± 62	71 ± 63	205 ± 178
Distance (%)	9 ± 9	10 ± 9	9 ± 8	10 ± 8
Time (s)	26 ± 27	26 ± 26	27 ± 25	80 ± 74
Time (%)	3 ± 3	3 ± 3	3 ± 2	3 ± 3
Individual workload (Kg·m)	4329 ± 4306	4395 ± 4029	4715 ± 4368	13439 ± 12019

Sprinting				
Distance (m)	6 ± 12	7 ± 13	7 ± 15	19 ± 36
Distance (%)	1 ± 1	1 ± 1	1 ± 1	1 ± 1
Time (s)	2 ± 4	2 ± 4	2 ± 4	5 ± 10
Time (%)	0.1 ± 0.3	0.1 ± 0.4	0.2 ± 0.5	0.1 ± 0.3
Individual workload (Kg·m)	342 ± 714	429 ± 899	480 ± 1102	1252 ± 2425

Total distance covered (m)	622 ± 131	631 ± 111	626 ± 137	1878 ± 333

Total individual workload (Kg·m)	40119 ± 8940	40695 ± 8465	40656 ± 10673	121471 ± 25652

The number of low, medium, high and total accelerations and decelerations is presented in Table 3. Total decelerations increased from the first to the second period (*p* = 0.039; *d* = 0.643).

**TABLE 3:** Number of accelerations and decelerations during each match period and total match duration of recreational team handball matches (mean ± SD)

Variables	1^st^ period	2^nd^ period	3^rd^ period	Total match
Low accelerations	6.1 ± 4.0	5.7 ± 3.5	5.8 ± 3.4	17.6 ± 10.4
Medium accelerations	3.8+ 2.2	3.8 ± 2.6	3.2 ± 2.1	10.8 ± 6.4
High accelerations	3.1+ 1.6	3.1 ± 2.2	3.1 ± 1.9	9.2 ± 5.1
Total accelerations	13.0 ± 6.9	12.6 ± 7.6	12.0 ± 6.7	37.6 ± 20.7

Low decelerations	3.5 ± 2.9	4.1 ± 2.9	3.7 ± 3.0	11.4 ± 8.5
Medium decelerations	1.4 ± 1.1	1.8 ± 1.1	1.5 ± 1.2	4.8 ± 3.1
High decelerations	0.9 ± 0.8	0.8 ± 0.6	0.7 ± 0.7	2.4 ± 1.7
Total decelerations	5.8 ± 4.4	6.7 ± 4.2^*^	6.0 ± 4.7	18.6 ± 13.0

* Significantly different from the first period.

Time spent in PL zones and total PL are present in Table 4. No differences were found in total PL between the match periods. Time spent in PL zone 0.1–0.3 (*p* = 0.015; *d* = 0.729) increased from the first to the third period, while time spend between 0.3–0.6 (*p* = 0.036; *d* = 0.630) and 1.0–1.5 (*p* = 0.010; *d* = 0.879) decreased from the first to the third period (Table 4). Time spent between 0.6–1.0 decreased over time (from the first to the second period: -1%; *p* = 0.036; *d* = 0.629; from the first to the third period: -2%; *p* < 0.001; *d* = 1.197).

**TABLE 4:** Time spent (%) in different Player Load zones and total Player Load during each match period and total match duration of recreational team handball matches (mean ± SD)

Variables	1^st^ period	2^nd^ period	3^rd^ period	Total match
**Player Load Zones**				
Time <0.1 (%)	25.28 ± 6.92	25.98 ± 7.40	27.67 ± 8.25	26.31 ± 6.45
Time 0.1-0.3 (%)	39.75 ± 6.93	41.37 ± 6.69	41.84 ± 6.36^*^	41.01 ± 6.39
Time 0.3-0.6 (%)	17.65 ± 4.65	17.19 ± 4.45	16.29 ± 5.10^*^	16.92 ± 4.59
Time 0.6-1.0 (%)	10.25 ± 3.37	9.24 ± 3.16^*^	8.33 ± 3.29^*^	9.26 ± 3.18
Time 1.0-1.5 (%)	5.80 ± 3.30	5.26 ± 2.79	4.76 ± 2.54^*^	5.31 ± 2.78
Time 1.5-2.0 (%)	1.04 ± 1.57	0.94 ± 1.38	0.93 ± 1.41	0.98 ± 1.42
Time >2.0 (%)	0.03 ± 0.15	0.07 ± 0.21	0.06 ± 0.15	0.06 ± 0.18

**Total Player Load (AU)**	77 ± 16	73 ± 14	72 ± 15	224 ± 41

* Significantly different from the first period.

The frequency of different game actions is described in Table 5. One-on-one situations decreased from first to the third period (*p* = 0.050; *d* = 0.714). No differences were found between the match periods in any other game actions.

**TABLE 5:** Game actions during each match period and total match duration of the recreational team handball matches (mean ± SD)

Actions	1^st^ period	2^nd^ period	3^rd^ period	Total match
Jumps	2.2 ± 2.2	1.9 ± 1.6	1.7 ± 1.3	5.9 ± 4.8
Throws	3.7 ± 2.7	3.5 ± 1.8	3.4 ± 1.9	10.6 ± 5.8
Stops	3.7 ± 1.3	3.7 ± 1.2	3.5 ± 1.6	11.0 ± 3.6
Changes of direction	2.5 ± 1.5	2.3 ± 1.2	2.1 ± 1.5	6.9 ± 3.9
One-on-one situations	1.4 ± 1.0	1.5 ± 1.4	0.9 ± 0.9^*^	3.8 ± 2.9

* Significantly different from the first period.

### Internal load reliability

HR variables’ absolute and relative reliability are presented in Table 6. Mean and peak HR presented good to moderate absolute reliability (TEM%CV ≤ 10%; CV% < 20%) and excellent relative reliability (ICC > 0.75). Time spent in the HR zones presented excellent relative reliability, except for time ≤ 60%HR_max_ and time 71–80%HR_max_, which was poor and good, respectively, and poor absolute reliability. RPE showed good relative reliability and poor absolute reliability.

**TABLE 6:** Reliability of heart rate variables (mean, peak and time spent in selected heart rate zones) and rating of perceived exertion across the three analyzed training sessions

Variables	ICC	95%CI	TEM	95%CI	TEM%CV	95%CI	CV%
Mean heart rate (b · min^−1^)	0.93	0.84–0.98	4.82	3.71–7.00	3.6	2.8–5.3	12.25
Mean heart rate (%HR_max_)	0.82	0.60–0.94	0.45	0.35–0.66	3.6	2.8–5.3	7.74
Peak heart rate (b · min^−1^)	0.95	0.87–0.98	4.34	3.34–6.31	2.9	2.2–4.2	11.06
Peak heart rate (%HR_max_)	0.84	0.62–0.94	2.54	1.96–3.70	2.9	2.2–4.2	6.39
Time ≤ 60%HR_max_ (%)	0.37	-0.02–0.71	2.11	1.62–3.06	67.9	49.6–116.8	153.4
Time 61–70%HR_max_ (%)	0.78	0.52–0.92	8.74	6.73–12.70	132.6	92.7–253.6	76.70
Time 71–80%HR_max_ (%)	0.74	0.46–0.90	0.54	0.41–0.78	101.4	71.5–177.8	77.31
Time 81–90%HR_max_ (%)	0.78	0.61–0.90	0.50	0.42–0.64	70.4	54.1–111.0	56.47
Time 91–100%HR_max_ (%)	0.82	0.59–0.93	11.11	8.56–16.16	135.9	93.2–254.1	113.99
Time > 80%HR_max_ (%)	0.77	0.51–0.92	0.51	0.39–0.74	69.9	50.9-120.9	57.93
RPE (AU; 0–10)	0.72	0.48–0.87	1.02	0.82–1.39	23.7	18.6–33.7	38.66

CI - Confidence interval; CV - coefficient of variance; ICC – Intraclass Correlation Coefficient; RPE – Rating of perceived exertion; TEM – Typical Error of the Measure.

## DISCUSSION

This is the first study analyzing the physiological and physical demands of small-sided RTH games, and the intensity variability of the training sessions (matches) in inactive 50–77-year-old women without prior experience with this sport. The main findings were that RTH is a high-intensity exercise modality, showing consistent results across the training sessions, with time spent in the HR zones presenting excellent relative reliability, though poor absolute reliability between the training sessions.

Mean HR values were > 77%HR_max_, which corresponds to vigorous PA [25] and almost half of the match time was spent in this intensity category. Mean HR increased from the first to the second period with high values being held across the 3 × 15-min periods. Additionally, time ≤ 60%HR_max_ (light-intensity) decreased after the first period. Thus, RTH is a vigorous-intensity exercise mode for postmenopausal women, able to be sustained over 45 min, and thereby can contribute for meeting the PA guidelines (volume and intensity). Nonetheless, the cardiovascular demands of RTH were lower for postmenopausal women than for younger populations [8–10, 14, 26], and were slightly higher than for 67-year-old men [15]. Moreover, there is consistency in mean and peak cardiovascular intensity across the training sessions, with high relative reliability, in spite of the absolute variability in the time spent in the HR zones. The collected data provide evidence for a satisfactory intra-individual consistency of the cardiovascular responses to RTH matches, despite the spontaneous nature of the considered exercise mode. However, it was evident that there is variability in the magnitude of the cardiovascular demands between training sessions, which suggests that if specific outcomes are targeted, such as a reaching a certain amount of time spent at a given cardiovascular intensity, a keen assessment and monitoring of the exercise intensity should be performed. Despite these findings, the resulting variability located the cardiorespiratory demands in the range considered helpful in producing sound health benefits in the participants [25], thus corroborating our hypothesis.

In this study, 14% of total match time was > 90%HR_max_. Time > 90%HR_max_ has been largely correlated with changes in cardiorespiratory fitness [7]. Accordingly, increases of 14%, 11% and 7% in cardiorespiratory fitness were reported for middle-aged men [7], young men [8] and postmenopausal women [12], involved in RTHbased exercise interventions, spending 21%, 14% and 11% of total match time > 90%HR_max_, respectively. Cardiorespiratory fitness is associated with reduced incidence of cardiovascular diseases and all-cause mortality [27], and consequently, any increase or even maintenance in $\dot{\text{V}}$O_2max_ is relevant. Thus, exercise modalities such as RTH that have the potential to improve $\dot{\text{V}}$O_2max_ are of the outmost importance. Nonetheless, even though postmenopausal women were shown to attain higher HRs and spend more time in higher intensity HR zones than older men, lower BL accumulation was found in comparison to their male counterparts (mean BL: 3.6–3.9 mmol · l^−1^; peak BL: 4.7–5.6 mmol · l^−1^) [15]. This is probably the result of a higher aerobic energy production and thus a lower anaerobic energy production of the considered female population during the RTH matches. However, comparison between BL concentrations across sexes should only be performed when normalizing for individual maximal values [28].

On the other side, this high-intensity physiological response contrasts with the amount of time spent in low-intensity locomotor categories. In fact, half of the total distance covered was performed walking and almost the total match time (92%) was spent standing or walking. Also in recreational football, high HRs were observed even when 70% of the match time was spent standing or walking, which suggests that other actions, besides the locomotor profile, could play a role in the match intensity [29]. Additionally, these findings highlight the relevance of HR or, in alternative, RPE being monitored in real time, to better assess and regulate exercise intensity. An increase was shown in decelerations from first to the second period and in time spent in low-intensity PL zone (0.1–0.3) from first to the third period, as well as a decrease in one-on-one situations. This decrement in external load demands was not in accordance with the high internal load demands during the matches, suggesting that external load variables pattern may not reflect changes in internal load, or vice versa, and highlights the importance of combining both measurements.

Despite all, a total of 38 accelerations (mainly low) and 19 decelerations were recorded. The number of accelerations, decelerations and total distance covered were correlated with changes in leg BMC of men with prostate cancer that participated in a recreational football exercise-based program for 12 weeks [30], being potential promoters of an osteogenic effect. Although, there are similarities between the physical demands of football and TH [31], the potential of these actions to induce skeletal muscle adaptations remains to be studied in TH. This would be of interest in future studies, as in fact, it has been previously showed that TH training results in positive bone adaptations [8, 10], namely in postmenopausal women [11]. Additionally, the frequency of different actions was also suggested as a contributor to the musculoskeletal adaptations shown in RTH players [14]. The number of actions performed by our participants was lower than that observed in other populations [8, 10, 15], which could be related, at least in part, to the inexperienced sport status of the population, and could have resulted in more ball losses and time-outs. Notwithstanding, RTH practice seems to offer a sufficient stimulus for postmenopausal women that increased lumbar spine BMD and BMC and femur BMC, and the expression of bone turnover markers after 16 weeks of practice [11].

The total distance covered by postmenopausal women was lower than that shown for younger populations playing RTH, namely young [26] and adult/middle-aged [14] men, that covered ~3 and ~6 km, respectively. However, the goalkeepers were not considered in the analysis of these studies, which can possibly explain the differences shown, as in our study the participants rotated positions, namely as outfield players and goalkeepers. Nonetheless, even when compared with 67-year-old men that covered ~5 km when playing RTH in 5 v 5 to 7 v 7 formats alternating between outfield and goalkeeper positions, postmenopausal women covered less than half the total distance [15]. The lower total distance covered per player in our study may have been the result of the lower area per player used in our study compared with the previous RTH mentioned studies (34–36 m^2^ per player vs. 57, 57–80 and 66–100 m^2^ per player [14],[15],[26]). In fact, an increase in the area per player resulted in an increase in the distance covered in recreational football players [32]. These particularities are of the most importance when the aim is to increase the external load imposed by exercise.

RPE was reported as 5.5 (hard; 0–10 AU), which is partly in accordance with other recreational team sport studies (range: 3.9–5.4) [26]. A higher RPE was expected, due to the vigorous intensity of the activity. Former TH players playing this sport recreational version reported a RPE of 7.6 (very hard) during RTH matches [14], which could reflect their ability to accurately evaluate the exercise intensity based on their training and competing experience. Nonetheless, a high level of fun (8.4, 0–10 AU) was reported by the participants. This finding is of the most importance, since lack of motivation is one of the main barriers to exercise, and thus RTH practice can potentially contribute for reducing the levels of physical inactivity shown in this population.

This study addresses the gap in the literature regarding the characterization of RTH in postmenopausal women without prior experience with the sport. It showed that exercise intensity is high and can be sustained during the total match duration (45 min), contributing to attain the PA guidelines. Moreover, due to its multicomponent nature, a feature recommended by PA guidelines, RTH-based programs can offer an alternative option to conventional exercise programs available for older populations. A strength of this study is the detailed characterization of the physical and physiological demands of RTH in this population, with the results supporting our study hypothesis, and also proving insights on the reasons that may explain the physical fitness, cardiovascular and musculoskeletal adaptations. Moreover, RTH showed to be a versatile exercise modality that can be played in different settings (indoor/outdoor), being thus adaptable to different contexts. Nonetheless, although there were no differences in internal load variables between indoor and outdoor conditions, it would be relevant to compare external load variables in these conditions, in order to better ascertain the physical characteristics of RTH practice in different settings. So, the lack of information about the external load demands in indoor conditions is a limitation of this study. Future studies should analyze the differences in the working demands of different game formats in this population, as well as for older populations with experience with the sport.

## CONCLUSIONS

Recreational TH training, played as small-sided games, is a highintensity exercise training mode, showing cardiovascular intensity consistency across training sessions with high aerobic and anaerobic demands, low RPE and high levels of fun for 50–77-year-old women without prior experience with the sport. These results are in line with previous studies using RTH with other populations and support the positive cardiorespiratory fitness, metabolic and musculoskeletal health effects of RTH practice shown in this population. Given the variability reported across matches, preliminary and or sustained activity demands control, i.e., HR monitoring and field testing, is suggested to optimize training responses.
